# A Cost-Effectiveness Analysis of “Test” versus “Treat” Patients Hospitalized with Suspected Influenza in Hong Kong

**DOI:** 10.1371/journal.pone.0033123

**Published:** 2012-03-29

**Authors:** Joyce H. S. You, Eva S. K. Chan, Maggie Y. K. Leung, Margaret Ip, Nelson L. S. Lee

**Affiliations:** 1 School of Pharmacy, Faculty of Medicine, Centre for Pharmacoeconomics Research, The Chinese University of Hong Kong, Shatin, Hong Kong, China; 2 Faculty of Medicine, Department of Microbiology, The Chinese University of Hong Kong, Shatin, Hong Kong, China; 3 Division of Infectious Diseases, Faculty of Medicine, Department of Medicine and Therapeutics, The Chinese University of Hong Kong, Shatin, Hong Kong, China; University of Liverpool, United Kingdom

## Abstract

**Background:**

Seasonal and 2009 H1N1 influenza viruses may cause severe diseases and result in excess hospitalization and mortality in the older and younger adults, respectively. Early antiviral treatment may improve clinical outcomes. We examined potential outcomes and costs of test-guided versus empirical treatment in patients hospitalized for suspected influenza in Hong Kong.

**Methods:**

We designed a decision tree to simulate potential outcomes of four management strategies in adults hospitalized for severe respiratory infection suspected of influenza: “immunofluorescence-assay” (IFA) or “polymerase-chain-reaction” (PCR)-guided oseltamivir treatment, “empirical treatment plus PCR” and “empirical treatment alone”. Model inputs were derived from literature. The average prevalence (11%) of influenza in 2010–2011 (58% being 2009 H1N1) among cases of respiratory infections was used in the base-case analysis. Primary outcome simulated was cost per quality-adjusted life-year (QALY) expected (ICER) from the Hong Kong healthcare providers' perspective.

**Results:**

In base-case analysis, “empirical treatment alone” was shown to be the most cost-effective strategy and dominated the other three options. Sensitivity analyses showed that “PCR-guided treatment” would dominate “empirical treatment alone” when the daily cost of oseltamivir exceeded USD18, or when influenza prevalence was <2.5% and the predominant circulating viruses were not 2009 H1N1. Using USD50,000 as the threshold of willingness-to-pay, “empirical treatment alone” and “PCR-guided treatment” were cost-effective 97% and 3% of time, respectively, in 10,000 Monte-Carlo simulations.

**Conclusions:**

During influenza epidemics, empirical antiviral treatment appears to be a cost-effective strategy in managing patients hospitalized with severe respiratory infection suspected of influenza, from the perspective of healthcare providers in Hong Kong.

## Introduction

Seasonal influenza results in excess hospitalization and mortality, with highest risk for young children, adults aged ≥65 years and patients with chronic medical conditions [1]. In 2009, a novel influenza A(H1N1) virus of swine origin had caused a pandemic [2]–[3]. The virus has continued to co-circulate with the seasonal influenza viruses in many parts of the world, in varying proportions. The key epidemiological feature of this novel infection is that younger adults <65 years are more commonly infected, and they too may develop severe and fatal diseases, even in the absence of underlying medical conditions [3]. Mortality rates of 2009 H1N1 influenza and seasonal influenza for hospitalized patients were estimated to be 2–15% and 3–8% respectively [4]–[6]. Most fatal cases of 2009 H1N1 influenza were aged 18–49 years [7].

A number of recent studies reported that early neuraminidase-inhibitor treatment within 48 hours of onset was associated with lower risks for disease progression and death in patients hospitalized with seasonal or 2009 H1N1 influenza [3], [6], [8]–[14]. Given these potential benefits, most health authority guidelines have suggested treatment for this patient group [2], [3]. A rapid clinical decision to treat hospitalized patients with suspected influenza is therefore important. However, clinical features of severe respiratory tract infection caused by influenza are indistinguishable from other viral or bacterial pathogens, and cannot be used to guide treatment (majority of hospitalized influenza patients do not present with the typical ‘influenza-like illness’) [3]. ‘Point-of-care’ rapid antigen tests for influenza are known to have very low sensitivities [3], [15]. More reliable rapid diagnostic assays, such as immunofluorescence assay (IFA) and Polymerase-Chain-Reaction (PCR), have been used to assist timely diagnosis and management of hospitalized patients [15]. For seasonal influenza, IFA has variable sensitivity (range 70–85%) but high specificity (99%); its sensitivity for 2009 H1N1 influenza is lower [16]–[18]. PCR is highly sensitive (>95%) and specific (98–100%) for influenza virus infection [19]–[23], but its use is often limited by the cost, as well as its availability and turn-around-time in some hospitals. On the other hand, the empirical antiviral treatment approach may offer timely therapeutic intervention to patients, yet exposes many more patients without influenza to antiviral agents. As there has been no consensus on the decision to ‘test’ or to ‘treat’ patients during influenza epidemics, we have conducted this analysis to evaluate the potential costs and outcomes of diagnostic test-guided and empirical antiviral treatment approaches in patients hospitalized for severe respiratory infection suspected of influenza, from the perspective of healthcare providers in Hong Kong.

## Materials and Methods

### Model Design

**Figure 1:**
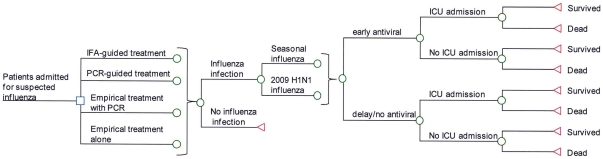
Simplified Decision Tree.

A decision tree was designed to simulate the outcomes of four clinical management strategies in a hypothetical cohort of adult patients hospitalized for severe respiratory infection, suspected of influenza, including: (1) using IFA, or (2) PCR testing to guide antiviral treatment; (3) empirical antiviral treatment plus PCR testing, and later decide to continue or discontinue treatment based on test results, and (4) empirical antiviral treatment alone (**Figure 1**). Three tiers of outcomes were simulated for each study arm: (1) total direct medical cost, (2) survival rate from influenza infection, and (3) quality-adjusted life-years (QALYs) expected. Case inclusion criteria were patients aged 18 years or above, had symptoms and signs compatible with influenza (e.g. fever, cough) and required hospitalization because of signs of severe lower respiratory infection: hypoxemia, tachypnea, and/or pulmonary infiltrates on chest radiography [8], [24].

In the present model, hospitalized patients with severe respiratory infection might or might not be infected with influenza virus. Furthermore, those who had influenza infection might present to the hospital within or beyond 48 hours from illness onset, and they might be infected by either a ‘seasonal’ virus or the 2009 H1N1 virus. In the ‘IFA-guided treatment” arm, patients with positive IFA test results would receive a course of oseltamivir. Given the low negative-predictive value of IFA, clinicians might still choose to treat despite a negative test result. In the “PCR-guided treatment” arm, patients with positive PCR results would receive a course of oseltamivir. Those who were test-negative would not receive treatment because of the high negative-predictive value of PCR. In the “empirical treatment plus PCR” arm, patients would receive empirical oseltamivir treatment and also tested for influenza viruses by PCR. Oseltamivir would be continued for the course if PCR result was positive, or discontinued the next day if the result was negative. In the “empirical treatment alone” arm, all patients would receive a full-course of oseltamivir. All patients who were infected with seasonal or 2009 H1N1 influenza viruses might survive or die, with or without being admitted to the Intensive Care Unit (ICU).

### Clinical Inputs

The clinical inputs of the model were shown in **Table 1**. A literature search on MEDLINE over the period of 2000–2011 was performed. The selection criteria of clinical studies of seasonal and 2009 H1N1 influenza were: (1) reports were written in English; (2) etiology of respiratory illnesses was identified to be seasonal or 2009 H1N1 influenza, and (3) mortality rate and/or ICU admission rate were reported. All articles retrieved by this process were screened for relevance to our model. A manuscript will be included if it had data pertaining to the model inputs.

**Table 1:** Model inputs.

	Base-case value	Range of sensitivity analysis	References
Clinical inputs			
Prevalence of influenza infections in admitted patients with suspected influenza	11%	0.1%–30%	[25]
Proportion of 2009 H1N1 influenza infections in admitted patients with influenza A infection	58%	0%–97%	[25]
Proportion of patients with influenza infections presented within 48 hours of onset	50%	0–100%	[14]
Susceptibility of influenza A viruses to oseltamivir	100%	99.3%–100%	[26]–[27]
ICU admission rate			
Seasonal influenza with late or no antiviral treatment	10.5%	3.1%–16.4%	[4], [14], [35]
Odds ratio with early antiviral treatment	0.93	0.46–1.8	[14]
2009 H1N1 influenza with late or no antiviral treatment	11.4%	10.5%–12.2%	[4], [36]
Odds ratio with early antiviral treatment	0.68	0.47–0.99	[36]
Morality rate			
Seasonal influenza with late or no antiviral treatment	4.9%	2.4%–10%	[4], [8], [14]
Odds ratio with early antiviral treatment	0.20	0.06–0.80	[8]–[9], [14]
2009 H1N1 influenza with late or no antiviral treatment	10%	2.2%–22.8%	[4]–[7], [10]–[11]
Odds ratio with early antiviral treatment	0.26	0.08–0.63	[10]–[11]
Sensitivity of diagnostic test			
IFA for seasonal influenza	79.2%	70%–85%	[16]–[17]
IFA for 2009 H1N1 influenza	50%	25%–75%	[18]
PCR for seasonal influenza	99%	98%–100%	[19]–[21]
PCR for 2009 H1N1 influenza	98%	86%–100%	[22]–[23]
Clinical judgment on influenza	44%	25%–75%	[37]
Specificity of diagnostic test			
IFA for influenza	99%	80%–100%	[16]–[17]
PCR for l influenza	97%	89%–100%	[19]–[21]
Clinical judgment on influenza	57%	25%–75%	[37]
Utility Inputs			
Utility score			
18–64 years	0.92	-	[28]
65–85 years	0.84	-	[28]
Mean age of patients hospitalized with seasonal influenza	70	18–80	[35], [38]
Mean age of patients hospitalized with 2009 H1N1 influenza	47	18–70	[5], [10]
Cost Inputs (USD)*			
Oseltamivir (per day)	6	5–9	-
Duration of oseltamivir treatment (days)	5	5–10	[39]
PCR	25	20–30	Expert opinion
IFA	10	5–10	Expert opinion
Hospitalization of influenza with no ICU care	7,957	17,955–26,932	[30]
Adjusting factor for cost of hospitalization with ICU care	5	4–6	-

* 1 USD = 7.8 HK.

**Table 2:** Results of base-case analysis on costs, survival event rates and QALYs expected from surviving influenza infections among hospitalized adults.

Strategy	Cost (USD)	Survival rate^a^	QALYs^b^	ICER^c^ (USD)
Empirical treatment alone	1,247	104.6	1.6917	-
PCR-guided treatment	1,248	104.5	1.6907	Dominated
IFA-guided treatment	1,249	103.8	1.6731	Dominated
Empirical treatment plus PCR	1,253	104.5	1.6907	Dominated

a : Survivals of influenza infection per 1,000 patients presented with suspected influenza.

b : Quality-adjusted life-years (QALYs) expected from patients infected with influenza A viruses.

c : ICER = increment cost per QALY gained.

The effectiveness of early antiviral treatment for seasonal and 2009 H1N1 influenza was estimated by the mortality rate, and the odds ratio of death associated with early antiviral treatment. Surveillance data on influenza activity in Hong Kong indicated that during 2010–2011, the prevalence of influenza A virus among all causes of respiratory tract infections ranged from 0.1% in the ‘low’ season to about 30% in the ‘peak’ season (i.e. percentage test positive among all clinical specimens obtained from symptomatic individuals) [25]. In base-case analysis, the average prevalence (11%) of influenza during the year was used to simulate the treatment outcomes; in addition, the impact of prevalence levels at low (0.1%) and peak (30%) seasons were examined in the sensitivity analysis. The proportion of 2009 H1N1 virus among all circulating influenza A viruses used in the base-case analysis (58%) was derived from the 2010–2011 surveillance data; this variable was examined over a wide range (0–97%) in the sensitivity analysis [25]. Surveillance on oseltamivir resistance among the influenza A viruses (seasonal strains and 2009 H1N1) isolated in Hong Kong during 2010–2011 showed that all such isolates were susceptible to oseltamivir [26]; about 0.7% of 2009 H1N1 viruses were reported to be resistant to oseltamivir in the literature [27]. Thus the model input for oseltamivir susceptibility was 100% for base-case analysis, and it was tested in the sensitivity analysis over the range of 99.3%–100%.

### Utility inputs

The QALYs expected by each influenza-infected patient was estimated from the age of patient and potential life-years expectancy surviving the infection. The utilities of adults aged 18–64 years and 65–85 years were retrieved from health-related quality of life scores reported in literature [28]. The future potential life-years gained were estimated using patient's age and life expectancy [29], and were discounted using a 3% discount rate per year.

### Cost Inputs

The economic analysis was conducted from the perspective of Hong Kong healthcare providers. A patient infected with influenza virus might be admitted to ICU, depending on the probability of ICU admission for seasonal or 2009 H1N1 influenza (and odds ratios of ICU admission after receiving early antiviral treatment). The model inputs for costs of managing respiratory infections were retrieved from our previous cost analysis of influenza with hospitalization [30]. The cost of ICU care versus non-ICU care was adjusted by a factor of 5 (ranging from 4–6), as daily cost of ICU care listed in the Hong Kong Gazette is approximately 5-fold of the daily cost of non-ICU care. The daily drug cost (USD5.8) of oseltamivir 75 mg twice daily therapy was retrieved from local retail pricing of oseltamivir. The treatment course of oseltamivir was 5 days in the base-case analysis (range 5–10 days). The costs (including reagents and manpower) of PCR and IFA with turn-around-time of less than 12 hours were derived from literature [31], and expert opinion. All costs were discounted to year 2011 costs with 3% discount rate.

### Cost-effectiveness analysis and Sensitivity Analysis

A treatment strategy was dominated when it was more costly and gained less QALYs than another treatment option. The incremental cost per QALY gained (ICER) of each arm (excluding the dominated strategy), comparing to the next less costly arm, was calculated using the following equation: Δcost/ΔQALYs. Using the threshold of USD50,000 as the willingness-to-pay per QALY [32], the most effective strategy with ICER USD50,000 or less was considered as cost-effective.

Sensitivity analysis was performed by TreeAge Pro 2009 (TreeAge Software, Inc., Williamstown, MA, USA) and Microsoft Excel 2007 (Microsoft Corporation, Redmond, WA, USA) to examine the robustness of the model results. All the parameters were examined over the upper and lower limits of the variables, if available. Otherwise, a range of variation by ±20% of the base-case value was used.

One-way sensitivity analysis on all variables was performed to screen for potential influential factors. To evaluate the impact of the uncertainty in all of the variables simultaneously, a probabilistic sensitivity analysis was performed using Monte Carlo simulation. The cost and QALYs of each study arm were recalculated 10,000 times by simultaneously varying the values of each model input through the ranges of sensitivity analysis to determine the percentage of time in which each study arm would be the most cost-effective option.

## Results

### Base-case analysis

In the base-case analysis (**Table 2**), it was shown that the QALY expected from surviving influenza infection in the “empirical treatment alone” study arm was highest (1.6917 QALYs), and that it was the least costly option (USD 1,247). The other three options including “IFA” (1.6731 QALYs, USD 1,249), “PCR-guided treatment” (1.6907 QALYs, USD 1,248) and “empirical treatment plus PCR” (1.6907 QALYs, USD 1,253) were all dominated by “empirical treatment alone”.

### Sensitivity analysis

**Figure 2:**
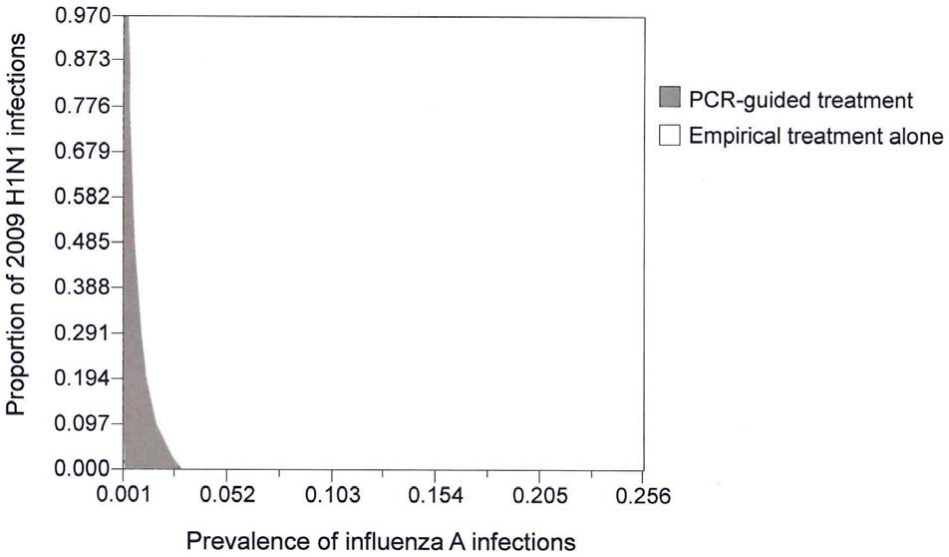
Two-way sensitivity analysis of prevalence of influenza and proportion of 2009 H1N1 infections on ICER per QALY expected by “empirical treatment alone” versus “PCR-guided treatment”.

The one-way sensitivity analysis had identified two influential model inputs on the ICER of “empirical treatment alone”: (1) prevalence of influenza in patients hospitalized for severe respiratory tract infections, and (2) proportion of 2009 H1N1 among all cases of influenza infections. A two-way sensitivity analysis was then conducted (**Figure 2**): “empirical treatment alone” was found to be the most cost-effective option in majority of the combinations of these two variables. Only when the prevalence of influenza was relatively low (<2.5%) and great majority of the circulating viruses were seasonal influenza strains, or when the influenza prevalence was extremely low (<0.4%) with high proportion of 2009 H1N1, the additional cost per QALY expected by “empirical treatment alone” would exceed USD50,000, and “PCR-guided treatment” would become the most cost-effective option with highest QALYs expected and additional cost per QALY less than USD50,000. All other options were dominated by either “empirical treatment alone” or “PCR-guided treatment” throughout the variations of these two variables in the model. We also examined the impact of the daily cost of antiviral treatment as it has been reported to be an influential factor on the cost-effectiveness of influenza treatment in literature [33]. The range of daily drug cost was extended and tested in one-way sensitivity analysis. It was found that “empirical treatment alone” would be dominated by “PCR-guided” when the daily cost of oseltamivir exceeded USD18.

**Figure 3:**
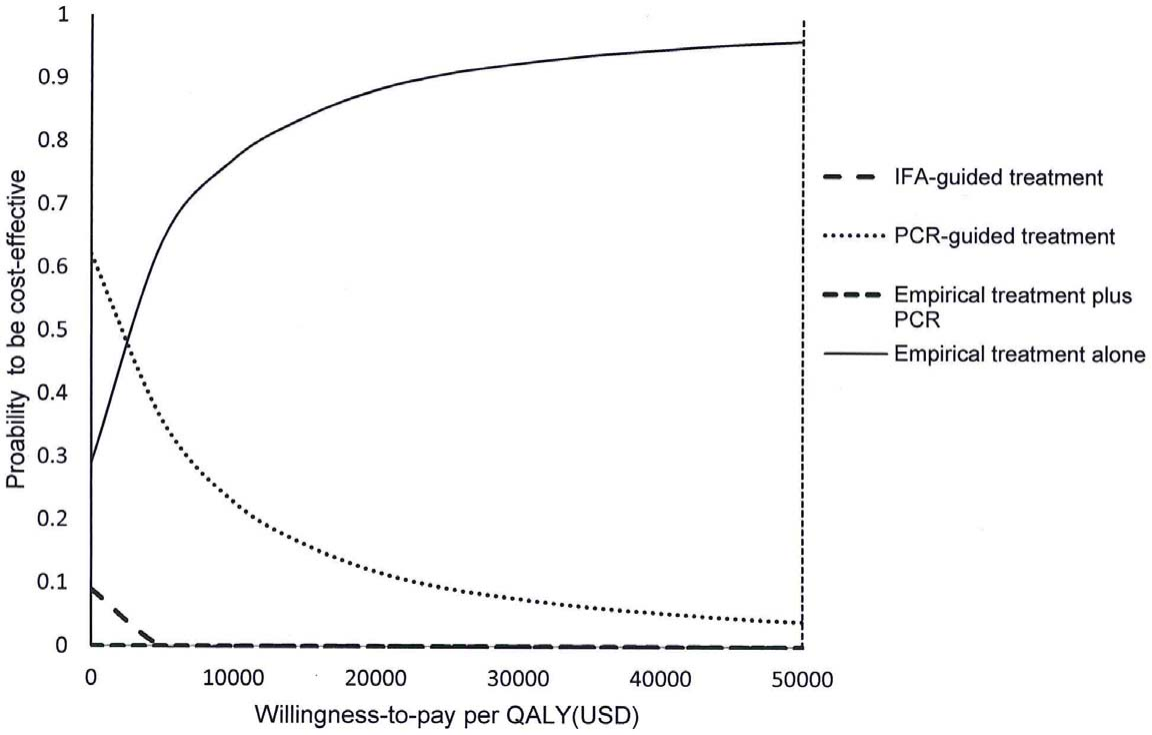
Acceptability curves of four treatment strategies to be cost-effective versus willingness-to-pay per QALY.

In the 10,000 Monte Carlo simulations generated by probabilistic sensitivity analysis, “empirical treatment alone” dominated the options of “IFA-guided treatment” and “empirical treatment plus PCR”. Comparing with the “PCR-guided treatment” option, “empirical treatment alone” was significantly more costly by USD4.9 (95%CI = 4.7–5.1), but with higher QALYs expected by 0.0029 QALYs (95%CI = 0.0028–0.0030) (p<0.001). The probabilities of each strategy to be cost-effective were examined in acceptability curves over a wide range of willingness-to-pay per QALY, from USD0-50,000 (**Figure 3**). Using USD50,000 as the threshold of willingness-to-pay, the probabilities of “empirical treatment alone” and “PCR-guided treatment” strategies to be most cost-effective were 97% and 3%, respectively.

## Discussion

The present study examined the cost-effectiveness of ‘empirical’ versus ‘test-guided’ influenza treatment strategies, at different levels of influenza prevalence and combinations of circulating viruses, in hospitalized adults with severe respiratory tract infection suspected of influenza. Our results suggested that in a season when the ‘seasonal influenza’ virus strains are predominant, “empirical antiviral treatment alone” would be a cost-effective option at influenza prevalence levels of 2.5% or above, whereas the ‘PCR-guided treatment’ approach would be cost-effective at a low prevalence of less than 2.5%. On the other hand, if 2009 H1N1 was predominating, ‘empirical treatment alone’ would be the more cost-effective option over a wide range of influenza prevalence levels (from 0.4% to >25%) as indicated by the two-way sensitivity analysis.

We found that in times of lower influenza prevalence (<2.5%), despite higher QALYs expected, the benefit of empirical treatment did not outweigh the cost of antivirals given to all hospitalized patients with respiratory infections suspected of influenza; the “PCR-guided treatment” approach was comparatively more cost-effective. However, when influenza prevalence increased to 2.5% or above, the empirical treatment approach would enable more patients to receive early therapeutic intervention within the first 48 hours of illness onset. The potential benefits of reduced ICU admissions and mortality among these hospitalized patients narrowed down the cost difference and increased the QALY gap between the two approaches, especially when 2009 H1N1 virus was the predominant circulating virus (which predominantly affected the younger adults). The robustness of empirical treatment being cost-effective was indicated by the Monte Carlo 10,000 simulations that the probability of “empirical treatment alone” to be cost-effective in 97% of suspected cases. The “IFA-guided treatment” was consistently dominated, which likely was the results of the relatively low diagnostic accuracy for influenza infections. “Empirical treatment plus PCR” was also dominated as a result of increasing treatment cost without significant change in QALYs, when compared with “PCR-guided treatment”.

Lee et al. had compared the cost-effectiveness of empirical intravenous (IV) peramivir alone, empirical peramivir plus PCR and PCR-guided peramivir for patients hospitalized with influenza-like illness and showed that PCR-guided treatment was most cost-effective, followed by empirical treatment alone. Empirical treatment plus PCR was found to be the least cost-effective option [33]. Different from their findings, our base-case analysis showed that empirical treatment alone was the more cost-effective option. It was likely due to the large cost difference between IV peramivir (USD20-1,000 per day) and oral oseltamivir (USD5.8 per day). In our one-way sensitivity analysis, the “PCR-guided treatment” strategy became the most cost-effective when the daily cost of oseltamivir was extended to USD18 and above, consistent with the findings of Lee et al. Our results also showed that “empirical treatment plus PCR” was less cost-effective than the “empirical treatment alone” and “PCR-guided treatment” options. Similar to our findings, empirical treatment was also more cost-effective than the diagnostic test-guided strategy for influenza in pediatric patients [34].

With new influenza virus strains emerging and increasing clinical evidence on antiviral treatment benefits, the management of patients hospitalized with influenza is evolving. Our decision analysis compared the potential changes in economic and clinical outcomes of four most commonly used management strategies in patients hospitalized with suspected influenza at a wide range of influenza prevalence levels, with varying combinations of circulating virus strains. The results suggested that “empirical treatment alone” is a cost-effective strategy in most situations during times of epidemics based on the key performance indexes (cost, QALYs, survival); however, the differences between the study arms are small and we acknowledge that clinical needs and practicability in individual settings are also important considerations. It should be emphasized that our results do not argue against the clinical use of laboratory tests for the management of individual patient hospitalized for severe influenza infection, or for the purpose of resistance monitoring [2]. Importantly, our decision model provides a framework to examine the influential factors and the corresponding threshold values (if any) for each strategy to translate into a cost-effective option. The present findings, in combination with real-time epidemiologic data through continuous surveillance, may assist the informed decision-making process of healthcare providers in future influenza seasons.

The present model was limited by sources of clinical model inputs which were mostly obtained from retrospective observational studies. The model inputs was therefore examined over a wide range in the sensitivity analyses to identify influential factors that would alter the base-case findings. In our analysis, we had assumed that a PCR assay's turn-around-time was less than 12 hours. However in many institutes, access to PCR could be limited and the results delayed for 1–2 days, or even longer. This could lower the QALYs gained and increase the total cost, further widening the cost and QALY differences between the comparative arms. The side-effects of oseltamivir are generally mild (e.g. GI intolerance), and therefore their impacts on cost and QALYs were not considered in the models. The variable of bacterial co-infection was also not included in the models for analysis due to the complexity of ‘community-acquired’ versus ‘hospital-acquired’ infections, and their highly variable prevalence and resistance profiles in different healthcare settings; also presence of bacterial co-infection should not alter the decision to initiate antivirals in influenza infection [7], [13]. The surveillance in Hong Kong showed that influenza A viruses are highly susceptible to oseltamivir (nearly 100%). Data from continuing surveillance should be used to update the impact of influenza viruses resistance on the decision analysis.

In conclusion, during influenza epidemics with prevalence >2.5%, empirical antiviral treatment appears to be a more cost-effective strategy in managing patients hospitalized with severe respiratory infection suspected of influenza, from the perspective of healthcare providers in Hong Kong.
